# ABO blood group system and risk of positive surgical margins in patients treated with robot-assisted radical prostatectomy: results in 1114 consecutive patients

**DOI:** 10.1007/s11701-021-01267-8

**Published:** 2021-06-29

**Authors:** Antonio Benito Porcaro, Nelia Amigoni, Filippo Migliorini, Riccardo Rizzetto, Alessandro Tafuri, Pierluigi Piccoli, Leone Tiso, Clara Cerrato, Alberto Bianchi, Sebastian Gallina, Rossella Orlando, Mario De Michele, Alessandra Gozzo, Stefano Zecchini Antoniolli, Vincenzo De Marco, Matteo Brunelli, Maria Angela Cerruto, Walter Artibani, Salvatore Siracusano, Alessandro Antonelli

**Affiliations:** 1 Department of Urology, Azienda Ospedaliera Universitaria Integrata Verona, University of Verona, Piazzale Stefani 1, 37126 Verona, Italy; 2 Chairman, Department of Urology, Azienda Ospedaliera Universitaria Integrata, University of Verona, Verona, Italy; 3 Department of Neuroscience, Imaging and Clinical Sciences, “G. D’Annunzio” University, Chieti, Italy; 4 Department of Transfusion Medicine, Azienda Ospedaliera Universitaria Integrata, University of Verona, Verona, Italy; 5 Department of Pathology, Azienda Ospedaliera Universitaria Integrata, University of Verona, Verona, Italy

**Keywords:** Prostate cancer, Robot-assisted radical prostatectomy, ABO blood system, Tumor load, Tumor stage, Positive surgical margins

## Abstract

To test the hypothesis of associations between the ABO blood group system (ABO-bg) and prostate cancer (PCa) features in the surgical specimen of patients treated with robot-assisted radical prostatectomy (RARP). Between January 2013 and October 2020, 1114 patients were treated with RARP. Associations of ABO-bg with specimen pathological features were evaluated by statistical methods. Overall, 305 patients were low risk (27.4%), 590 intermediate risk (50%) and 219 high risk (19.6%). Pelvic lymph node dissection was performed in 678 subjects (60.9%) of whom 79 (11.7%) had cancer invasion. In the surgical specimen, tumor extended beyond the capsule in 9.8% and invaded seminal vesicles in 11.8% of cases. Positive surgical margins (PSM) were detected in 271 cases (24.3%). The most frequently detected blood groups were A and O, which were equally distributed for both including 467 patients (41.9%), followed by groups B (127 cases; 11.4%) and AB (53 subjects; 4.8%). Among specimen factors, the ABO-bgs associated only with the risk of PSM, which was higher for blood group O (30.4%) compared with group A (19.5%) after adjusting for other standard clinical predictors (odds ratio, OR = 1.842; 95% CI 1.352–2.509; *p* < 0.0001). Along the ABO-bgs, the risk of PSM was increased by group O independently by other standard preoperative factors. The ABO-bgs may represent a further physical factor for clinical assessment of PCa patients, but confirmatory studies are required.

## Introduction

Actually, prostate cancer (PCa) is one of the most investigated cancers in the aging male who is likely to have the disease detected at early stages [1, 2]. In early PCa, several management options are proposed, which include active surveillance, primary radiation, and radical prostatectomy (RP), which may be performed by the open approach (ORP) or more frequently by the robot-assisted procedure (RARP), as well [1, 2]. However, clinical PCa includes a heterogeneous set of patients who are classified into risk categories by prognostic clinical factors including prostate-specific antigen (PSA), tumor stage, and grade, as well [1, 2]. In the surgical specimen, tumor upgrading and upstaging as well as the detection of positive surgical margins (PSM) are unfavorable outcomes requiring further management decisions. So far, aggressive PCa biology may be detected in the surgical specimen after RARP; as such, further clinical factors are required to stratify risk categories [1, 2].

Potential preoperative factors for stratifying PCa clinical risk classes could be represented by blood group antigens, which are polymorphic, inherited structural characters that are present on the outer surface of the red cell membrane and are located on proteins, glycoproteins or glycolipids; furthermore, human blood group antigens have also been associated with clinical disorders[3]. The ABO blood group system (ABO-bgs) is the most important not only for being the first one discovered but also for both blood transfusions and organ transplantation; furthermore, it associates with non-oncological and oncological diseases. In case–control studies, the ABO-bgs has been associated with the risk of several epithelial cancers [4–10]. For example, the risk of gastric cancer was increased by blood group A while individual belonging to the non-O-bgs showed an increased risk of pancreatic cancer, as well [4–7]. Recently, associations between phenotype ABO-bgs and prostate cancer have been hypothesized by a case–control study that did not show any significant association [11]. However, a retrospective study, which investigated a small heterogeneous cohort of PCa patients, has shown associations between high-risk PCa and ABO-bgs [12]. Furthermore, another retrospective study showed that the ABO-bgs correlated with survival on PCa vaccine therapy [13]. So far, the hypothesis of associations between ABO-bgs and PCa is an unsettled topic that needs more clinical investigations, which should be interpreted according to the complexity of clinical and pathological manifestations of PCa [14].

In this study, we wanted to investigate associations between the ABO-bgs and PCa features in the surgical specimen of patients treated with RARP.

## Materials and methods

### Study population

The study was retrospective and approved by the internal Institutional Review Board. Informed signed consent was obtained by all patients. Data were collected prospectively but evaluated retrospectively. In a period ranging from January 2013 to October 2020, 1114 consecutive patients who underwent RARP were included after excluding cases who were under androgen blockade and/or had prior treatments for PCa. Surgical procedures were performed by five skilled and dedicated surgeons of whom two were classified as high volume. Clinical features including age (years), body mass index (BMI; kg/m^2^), PSA (ng/mL), prostate volume (PV, mL) and biopsy positive cores (BPC; %) were evaluated. Tumors were staged according to clinical and pathological TNM system [1, 2]. RARP was eventually associated with pelvic lymph node dissection (PLND) according to guideline recommendations or tumor upgrading probability for the low-risk category [15, 16]. Lymph node dissection was developed according to a standard anatomical template including external iliac, obturator, Cloquet’s and Marcille’s regions [17, 18]. Since January 2017, our policy is not to place a drain in the pelvic cavity independently by performing or not an extended PLND [19].

Operations were performed by surgeons who were classified into high- and low-volume (> 100) according to study reporting a reduction PSM rate after 100 cases were performed [20]. Specimens were evaluated for tumor grade and stage, surgical margins, number of removed, and metastatic lymph nodes. Tumors were graded according to the International Society of Urological Pathology (ISUP) system [1, 2]. Preoperative surgical risk was evaluated by the American Society of Anesthesiologists (ASA) score system [21]. Postoperative surgical complications were graded according to the Clavien–Dindo system [1, 2]. At hospital discharge, patients were followed for a period of 90 days to detect complications and/or hospital readmission events. In each patient, the genotype ABO blood group system was assessed preoperatively by the Department of Transfusion Medicine. Blood groups were routinely determined on microplates by reactant and instrumentation LIFE (AstraFormedic, Gruppo De Mori).

### Statistical methods

The hypothesis of associations between the ABO-bgs and PCa biology was tested on specimen pathological features. According to their distributions, continuous variables were represented as medians (interquartile range, IQR) while categorical variables were assessed as frequencies (percentages). The association of the ABO-bg system with clinical and pathological variables was assessed by the multinomial logistic regression model (univariate analysis). The independent association of the ABO-bgs with specimen pathological features was eventually assessed by the logistic regression model (univariate and multivariate analysis). The fit of potential multivariate models including the ABO-bgs after adjusting for PCa clinical features was assessed the Hosmer–Lemeshow test, which was performed after computing the associated decile contingency tables. The software used to run the analysis was IBM-SPSS version 26. All tests were two-sided with *p* < 0.05 considered to indicate statistical significance.

## Results

### Demographics and cancer features of the patient population

Table 1 shows the demographics of the patient population that included 1114 cases of whom 305 were low-risk (27.4%), 590 intermediate-risk (50%) and 219 high-risk (19.6%). Pelvic lymph node dissection was performed in 678 subjects (60.9%) of whom 79 (11.7%) had cancer invasion. In the surgical specimen, tumor extended beyond the gland in 240 patients (21.6%) with extracapsular extension in 9.8% and seminal vesicle invasion in 11.8% of cases, respectively; furthermore, surgical margins resulted involved by cancer in 271 cases (24.3%). PMS location was at apex in 41% of cases and in 34% at posterolateral base gland (left or right).

**Table 1:** Demographics of the prostate cancer population (*n* = 1114) that was treated with robot-assisted radical prostatectomy (RARP)

	Median (IQR) or frequency (%)
Clinical factors	
Age (years)	65 (61–70)
Body mass index, BMI (kg/m^2^)	25.7 (23.9–28)
Prostate specific antigen, PSA (μg/L)	7 (5.1–9.7)
Prostate volume, PV (mL)	40 (30.3–52)
Biopsy positive cores, BPC (%)	34.5 (21–53)
International Society of Urologic Pathology (ISUP) tumor grade system	
ISUP = 1	436 (39.1)
ISUP = 2	356 (32.0)
ISUP = 3	192 (17.2)
ISUP = 4	106 (9.5)
ISUP = 5	24 (2.2)
Tumor clinical stage (cT)	
cT1	687 (61.7)
cT2/3	427 (38.3)
Clinical nodal stage (cN)	
cN0	1058 (95)
cN1	56 (5)
American Society of Anesthesiologists’ (ASA) physical system	
ASA I	104 (9.3)
ASA II	905 (81.2)
ASA III	105 (9.5)
D’Amico risk groups	
Low risk class	305 (27.4)
Intermediate risk class	590 (53.0)
High risk class	219 (19.6)
Pathological factors	
Prostate weight; gr (PW)	51 (42–65)
ISUP = 1	143 (12.8)
ISUP = 2	438 (39.3)
ISUP = 3	303 (27.2)
ISUP = 4	158 (14.2)
ISUP = 5	72 (6.5)
Pathological tumor stage (pT)	
pT2	874 (78.5)
pT3a	109 (9.8)
pT3b	131 (11.8)
Positive surgical margin (PSM)	
No	843 (75.7)
Yes	271 (24.3)
Pathological nodal staging (pN)	
pN0	599 (53.8)
pN1	79 (7.1)
pNx	436 (39.1)
Lymph nodes removed (number)	25 (20–32)
Perioperative factors	
High volume surgeon (HVS)	600 (53.9)
Low volume surgeon (LVS)	475 (42.6)
Unknown	39 (3.5)
Operating time; min (OT)	233 (205–259.3)
Blood lost; mL (BL)	300 (150–400)
Any post-operative Clavien–Dindo complication at discharge (CDC)	273 (24.5)
Length of hospital stay; days (LOHS)	4 (4–5)
Hospital readmission; *n* (%)	35 (3.1)

*IQR* interquartile range, *%* percentage

**Fig. 1:**
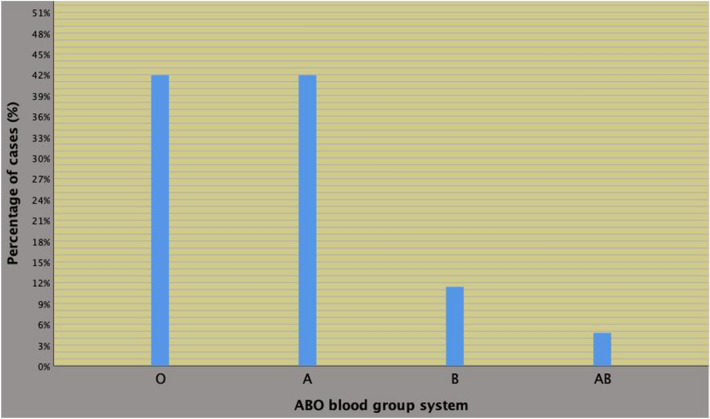
Distribution of the ABO blood group system in 1114 prostate cancer (PCa) subjects who underwent robot-assisted radical prostatectomy (RARP). As shown, the most frequently detected blood groups were A and O, which were equally distributed for both being detected in 467 patients (41.9%), followed by groups B (127 cases; 11.4%) and AB (53 subjects; 4.8%)

Preoperative physical status included prevalently ASA grade group II (905 cases; 81.2%) whereas groups I and III almost overlapped for including 104 (9.3%) and 105 (9.5%) cases, respectively. As shown in Fig. 1, the most frequently detected blood groups were A and O, which were equally distributed for both being detected in 467 patients (41.9%); furthermore, groups B (127 cases; 11.4%) and AB (53 subjects; 4.8%) then followed. Other clinical and perioperative features are detailed in the referred table.

Most part of the procedures was performed by high-volume surgeons and no significant differences in PSM rate were found according to surgeon experience.

### Associations of ABO-bg system with specimen pathological features

**Fig. 2:**
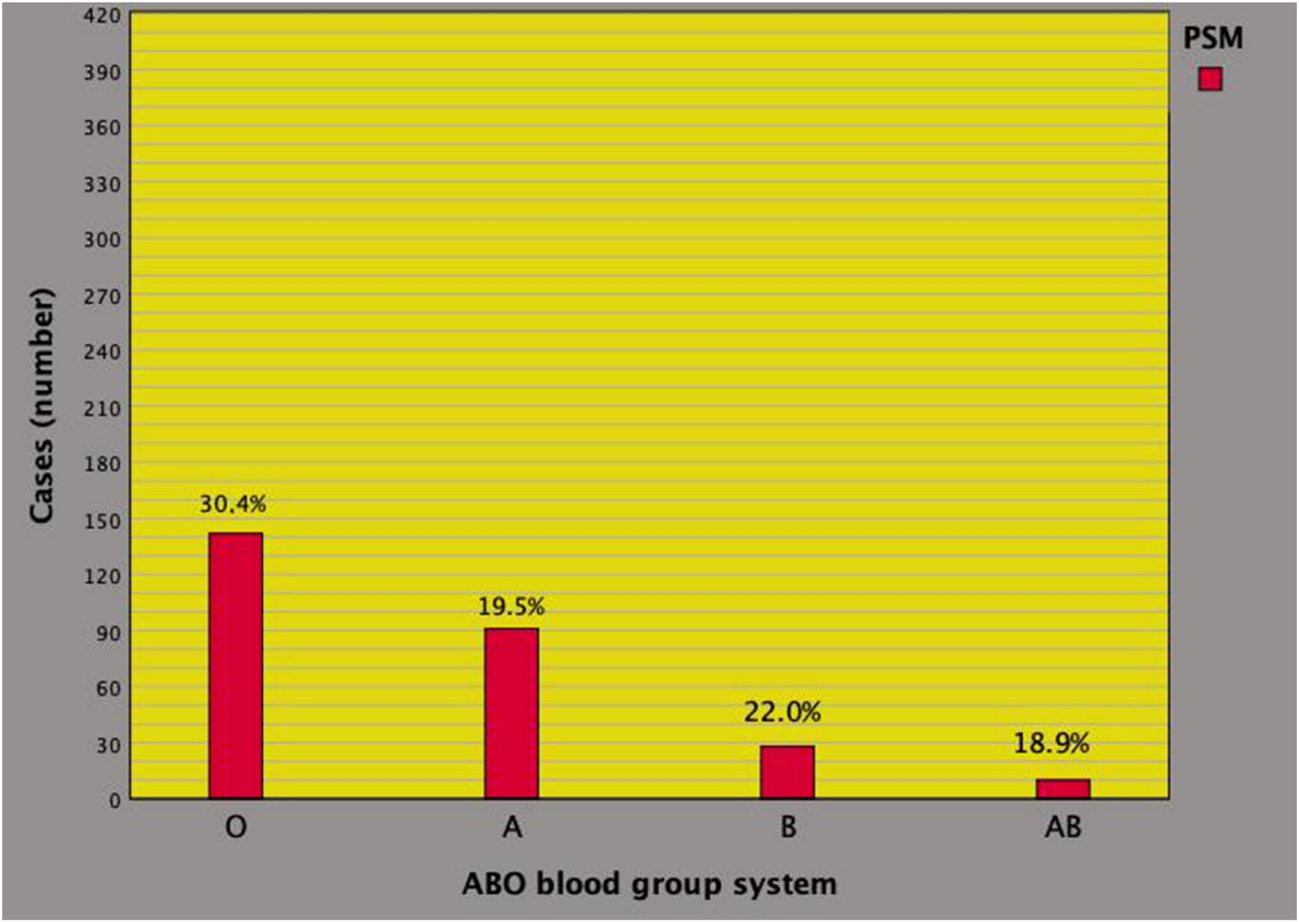
Distribution of positive surgical margins (PSM) along the ABO blood group system in the surgical specimen of 1114 consecutive prostate cancer (PCa) who were treated with robot-assisted radical prostatectomy (RARP). As illustrated, the distribution of cases was as follows: 142 (30.4%) for group O, 91 (19.5%) for group A, 28 (22%) for group B, and 10 (18.9%) for group AB

Table 2 summarizes the results of potential associations of the ABO-bg with clinical, pathological, and perioperative features of the investigated patient population. For the overlapping distribution of the two main systems, blood groups O, B, and AB were compared with group A. Overall, the risk of detecting PSM was significantly higher for blood group O compared with group A (odds ratio, OR = 1.805; 95% CI 1.355–2.442; *p* < 0.0001). Figure 2 depicts the distribution of PSM in each group. Excluding age, which was inversely related in blood group B when compared with group A, no other significant associations were detected.

**Table 2:** Associations of clinical, pathological, and perioperative factors with the ABO blood group system in 1114 prostate cancers patients treated with robot assisted radical prostatectomy (univariate analysis)

Statistics	Blood group O vs A		Blood group B vs A		Blood group AB vs A	
	OR (95% CI)	*p* value	OR (95% CI)	*p* value	OR (95% CI)	*p* value
Age	0.984 (0.965–1.004)	0.115	0.968 (0.940–0.997)	0.033	0.979 (0.938–1.022)	0.331
BMI	1.011 (0.970–1.053)	0.610	1.015 (0.954–1.079)	0.648	0.926 (0.844–1.017)	0.109
PSA	1.006 (0.989–1.024)	0.484	1.000 (0.972–1.029)	1.000	1.000 (0.960–1.042)	1.000
PV	0.997 (0.990–1.004)	0.431	0.997 (0.986–1.008)	0.586	0.999 (0.984–1.015)	0.929
BPC	0.998 (0.992–1.004)	0.522	0.998 (0.989–1.008)	0.747	0.997 (0.984–1.010)	0.647
ISUP < 3	Ref		Ref		Ref	
ISUP > 2	0.969 (0.730–1.287)	0.828	1.001 (0.650–1.541)	0.998	1.052 (0.556–1.955)	0.871
cT < 2	Ref		Ref		Ref	
cT > 1	0.904 (0.694–1.179)	0.904	1.070 (0.717–1.597)	0.741	1.656 (0.937–2.927)	1.656
cN0	Ref		Ref		Ref	
cN1	1.371 (0.758–2.482)	0.296	1.108 (0.435–2.821)	0.829	1.341 (0.385–4.672)	0.645
PW	0.998 (0.991–1.005)	0.559	0.996 (0.986–1.007)	0.985	1.006 (0.993–1.020)	0.365
ISUP < 3	Ref		Ref		Ref	
ISUP > 2	1.118 (0.865–1.445)	0.395	0.930 (0.625–1.379)	0.718	1.186 (0.672–2.094)	0.556
pT2	Ref		Ref		Ref	
pT3a	1.099 (0.714–1.693)	0.667	0.781 (0.381–1.602)	0.500	1.171 (0.472–2.909)	0.733
pT3b	1.186 (0.799–1.762)	0.397	0.796 (0.410–1.544)	0.499	0.829 (0.314–2.188)	0.705
No PSM	Ref		Ref		Ref	
PSM	1.805 (1.355–2.442)	< 0.0001	1.169 (0.725–1.885)	0.523	0.961 (0.465–1.984)	0.914
pN0	Ref		Ref		Ref	
pN1	0.918 (0.542–1.555)	0.751	1.328 (0.617–2.858)	0.468	2.425 (0.962–6.114)	0.060
LN (*n*)	1.003 (0.987–1.019)	0.750	1.015 (0.991–1.040)	0.231	0.994 (0.957–1.034)	0.776
No PLND	Ref		Ref		Ref	
PLND	1.201 (0.921–1.556)	0.177	0.806 (0.542–1.197)	0.285	0.856 (0.482–1.519)	0.595
OT	1.001 (0.999–1.003)	0.362	0.998 (0.994–1.002)	0.269	0.999 (0.994–1.004)	0.667
BL	1.000 (1.000–1.000)	0.715	1.000 (0.999–1.000)	0.476	1.000 (0.998–1.001)	0.425
CDS = 0	Ref		Ref		Ref	
CDS > 0	1.123 (0.033–1.514)	0.446	1.201 (0.768–1.878)	0.423	0.860 (0.428–1.728)	0.672
LOHS	1.039 (0.971–1.111)	0.265	0.918 (0.800–1.052)	0.219	1.048 (0.918–1.198)	0.488
No RAD	Ref		Ref		Ref	
RAD	1.258 (0.583–2.718)	0.559	1.880 (0.691–5.112)	0.216	1.487 (0.324–6.830)	0.610

See also Table 1

*OR* odds ratio, *CI* confidence interval

### The ABO-bg system as an independent predictor of the risk of PSM

On multivariate analysis, the risk of PSM still associated with blood group O when compared with group A (odds ratio, OR = 1.820; 95% CI 1.344–2.464; *p* < 0.0001) independently by BMI (inverse association) for physical factors at clinical presentation, as shown in Table 3. As expected, the risk of PSM also associated with clinical and pathological PCa features. The risk of PSM increased as PSA, BPC, pT and pathological tumor grade increased as well decreased as TV increased. Table 4 shows the risk of PSM as predicted by preoperative multivariate models including the ABO-bgs, which was adjusted for BMI in model I as well as for PCa features in model II (PSA, BPC, PV). The risk of detecting PSM in the surgical specimen was higher for blood group O (30.4%) compared with group A (19.5%) with the former increasing the predictive power from model I (OR = 1.820; 95% CI 1.344–2.464; *p* < 0.0001) to model II (OR = 1.842; 95% CI 1.352–2.509; *p* < 0.0001), as shown by the Hosmer–Lemeshow test and associated contingency tables.

**Table 3:** Associations of physical, cancer and perioperative factors with the risk of positive surgical margins (PSM) in 1114 prostate cancer patients treated with robot assisted radical prostatectomy (RARP)

	NSM	PSM	PSM vs NSM		PSM vs NSM	
	Statistics		Univariate analysis		Multivariate analysis	
	Median (IQR) or frequency (%)	Median (IQR) or frequency (%)	OR (95% CI)	*p* value	OR (95% CI)	*p* value
*N* (%)	843 (75.7)	271 (24.3)				
Physical factors						
Blood group						
A	376 (44.6)	91 (33.6)	Ref		Ref	
B	99 (11.7)	28 (10.3)	1.169 (0.725–1.885)	0.523	1.175 (0.728–1.1898)	0.51
AB	43 (5.1)	10 (3.7)	0.961 (0.465–1.984)	0.914	0.922 (0.445–1.909)	0.83
O	325 (38.6)	142 (52.4)	1.805 (1.335–2.442)	< 0.0001	1.820 (1.344–2.464)	< 0.0001
Age	65 (61–70)	66 (61–71)	1.003 (0.982–1.024)	0.807		
BMI	25.9 (24–28)	25.3 (23.1–28)	0.950 (0.909–0.993)	0.023	0.947 (0.905–0.990)	0.02
ASA I-II	760 (90.2)	249 (91.9)	Ref			
ASA III	83 (9.8)	22 (8.1)	0.809 (0.495–1.322)	0.398		
Cancer clinical factors						
PSA	6.7 (5–9)	7.8 (5.4–12.2)	1.044 (1.021–1.067)	< 0.0001	1.042 (1.108–1.066)	0
PV	40 (31–53)	40 (30–50)	0.989 (0.981–0.998)	0.013	0.988 (0.979–0.996)	0.01
BPC	33 (21–50)	42 (28.3–63.5)	1.013 (1.007–1.019)	< 0.0001	1.009 (1.003–1.016)	0.01
ISUP < 3	607 (72)	185 (68.3)	Ref			
ISUP > 2	236 (28)	86 (31.7)	1.196 (0.889–1.609)	0.238		
cT < 2	528 (62.6)	159 (58.7)	Ref			
cT > 1	315 (37.4)	112 (41.3)	1.181 (0.893–1.561)	0.243		
cN0	802 (95.1)	256 (94.5)	Ref			
cN1	41 (4.9)	15 (5.5)	1.146 (0.624–2.105)	0.660		
Cancer specimen factors						
PW	52 (43–65)	50 (41–62.5)	0.991 (0.984–0.999)	0.028	0.995 (0.985–1.004)	0.29
ISUP < 3	472 (56)	109 (40.2)	Ref		Ref	
ISUP > 2	371 (44)	162 (59.8)	1.891 (1.432–2.498)	< 0.0001	1.678 (1.105–2.548)	0.02
pT2	704 (83.5)	170 (62.7)	Ref		Ref	
pT3a	64 (7.6)	45 (16.6)	2.912 (1.920–4.416)	< 0.0001	2.259 (1.334–3.827)	0
pT3b	75 (8.9)	56 (20.7)	3.092 (2.105–4.542)	< 0.0001	2.072 (1.256–3.419)	0
pN0	456 (91.0)	143 (80.8)	Ref		Ref	
pN1	45 (9.0)	34 (19.2)	2.409 (1.486–3.907)	< 0.0001	1.494 (0.859–2.5979	0.16
LN (*n*)	25 (20–32)	25 (21–31.5)	0.996 (0.979–1.013)	0.661		
Perioperative factors						
No PLND			Ref		Ref	
PLND			1.302 (0.978–1.735)	0.071		
HVS	477 (57.9)	123 (49.0)	Ref			
LVS	347 (42.1)	128 (51)	1.431 (1.077–1.899)	0.013	1.290 (0.947–1.758)	0.11
OT	230 (200.5–255)	244 (215–273)	1.004 (1.001–1.006)	0.003	1.002 (0.999–1.005)	0.2
BL	250 (150–400)	300 (200–500)	1.001 (1.000–1.001)	0.009	1.001 (1.000–1.001)	0.01
CDS = 0	645 (76.5)	196 (72.3)	Ref			
CDS > 0	198 (23.5)	75 (27.7)	1.247 (0.914–1.700)	0.164		
LOHS	4 (4–5)	4 (4–5)	1.057 (0.990–1.130)	0.097		
No RAD	816 (96.8)	263 (97.0)	Ref			
RAD	27 (3.2)	8 (3.0)	0.919 (0.413–2.048)	0.837		

See also Table 1

*NSM* negative surgical margins, *IQR* interquartile range, *OR* odds ratio, *CI* confidence interval

**Table 4:** Multivariate clinical models of ABO blood group system predicting the risk of positive surgical margins (SM) in 1114 prostate cancer patients treated with robot-assisted radical prostatectomy (RARP)

Multivariate model							
	Total	NSM	PSM	PSM vs NSM; Model I*		PSM vs NSM; Model II**	
	*n*	*n* (%)	*n* (%)	OR (95% CI)	*p* value	OR (95% CI)	*p* value
Blood group system							
A	467	376 (80.5)	91 (19.5)	Ref		Ref	
O	467	325 (69.6)	142 (30.4)	1.820 (1.344–2.464)	< 0.0001	1.842 (1.352–2.509)	< 0.0001

Assessing the fit of	Model I					Model II				
Group	Total	NSM		PSM		Total	NSM		PSM	
		Observed	Predicted	Observed	Predicted		Observed	Predicted	Observed	Predicted
1	112	92	94.3	20	17.7	111	100	98	11	13
2	113	93	92.6	20	20.4	111	93	93.9	18	17.1
3	110	84	88.7	26	21.3	111	91	91.8	20	19.2
4	108	86	85.7	22	22.3	111	97	89.7	14	21.3
5	111	94	86.7	17	24.3	111	82	87.1	29	23.9
6	110	82	83.6	28	26.4	111	87	84.6	24	26.4
7	116	82	84.5	34	31.5	111	83	81.9	28	29.1
8	111	83	78.2	28	32.4	111	76	78.5	35	32.3
9	111	83	75.7	28	35.3	111	72	74.2	39	36.8
10	112	64	72.9	48	39.1	115	62	63.3	53	51.7
Total	1114					1114				

See also Table 1. Test of Hosmer–Lemeshow: (a) Model I: Chi-squared 11.109; degree freedom = 8; *p* = 0.196, overall accuracy 75.7%; (b) Model II: Chi-squared 5.842, degree freedom = 8, *p* = 0.665, overall accuracy 76.1%

*NSM* negative SM, *PSM* positive SM, *OR* odds ratio, *CI* confidence interval

*Model adjusted for blood group B, blood group AB and BMI

**Model adjusted for blood group B, blood group AB, BMI, PSA, PV and BPC

## Discussion

The ABO-bgs is traced out by the ABO gene, which is single and located on chromosome 9q34; furthermore, it still remains the most important system for both transfusion and transplantation medicine [3, 4]. The ABO-bgs has been associated with the risk of several carcinomas such as stomach, pancreas, ovary, kidney, and skin [4–12]. Specifically, it has been demonstrated that genotype blood group non-O increased the risk of cancers involving pancreas, kidney, and ovary but not non-melanoma skin cancer, which was instead increased by group O; furthermore, gastric cancer was the first malignant tumor that associated with phenotype A-bgs [3–12]. So far, several studies show associations between the ABO-bgs and epithelial cancers. So far, a potential association between the ABO-bgs and PCa could be supposed. Indeed, PCa shows complex clinical and pathological manifestations that should be considered when planning and analyzing clinical studies [14]. Potential associations of the ABO-bgs with PCa represents a new topic, which is actually in progress. In a large case–control study, Iodice et al. did not show any significant association between the ABO-bgs and risk of epithelial cancers, which also included PCa; furthermore, in that trial, the distribution of the ABO-bg between controls vs PCa case was 46% vs 42% for group O, 42% vs 43% for group A, 9% vs 10% for group B and 3% vs 4% for group AB [4]. Markt et al. did not find any significant association between ABO-bgs and risk of aggressive PCa or PCa specific mortality in a large case–control study, which was restricted to men of European ancestry, including 2774 aggressive PCa cases and 4443 controls; furthermore, the distribution of the ABO-bgs for controls versus cases was 42% vs 40% for group O, 43% vs 44% for group A, 10% vs 12% for group B and 5% vs 5% for group AB [11]. Multhana et al., in a retrospective analysis of prospective phase II trial on immunotherapies in PCa (PROSTVAC-VF), showed longer median survival in patients with blood type B and O compared with groups A and AB [13]. Wang et al., in a single-center study conducted on the Chinese PCa population, showed that the risk of aggressive PCa was higher for non-O blood groups compared with group O; however, the trial had several limitations for being retrospective, for the size of the sample, for the definition of “high-risk” patients who were widely heterogenous for being at the same time high-risk, locally advanced or even metastatic; furthermore, the low-middle risk subpopulation included only 43 (18.1%); as such, the results of the study are difficult to apply to the Caucasian population [12]. In a Caucasian PCa population treated with RARP, we found out an independent association between the ABO-bgs and risk of PSM in the surgical specimen. Specifically, the risk of detecting a PSM was higher for blood group O when compared with group A, independently by physical (BMI) and cancer clinical features (PSA, BPC and PV). We have also shown that the ABO-bgs was an effective predictor of PSM after adjusting for physical and cancer preoperative factors thus demonstrating close association with PCa biology. As such, the results of our study represent a novelty in the literature dealing with this subject and might have implications in clinical practice.

Actually, the prevalence of PSM after RP ranges from 8.8 to 37%; and independent predictors are represented by surgeon’s volume and tumor biology including features related to load, extension and aggressiveness of cancer [22–24]. In tertiary referral centers, RARP is the most prevalent procedure and it decreases the risk of PSM when compared with ORP; as a result, these outcomes further support the advantages of oncological outcomes of robotic surgery [25]. We did not find difference in other clinical pathological features among ABO-bgs groups. However, we have confirmed that clinical and pathological features related to the risk of PSM, which increased as PSA, BPC, tumor upstaging, and upgrading after surgery. The small number of cases including blood groups B and AB may explain the missed association with the risk of PSM for these subgroups. Additionally, no differences were found according to surgeons’ experience in the PSM rate. This data could be influenced by the high number of procedures performed by high-volume surgeons compared to low-volume surgeons, and maybe in contrast with our previous experience where we included only one high-volume surgeon according to the department organization referred to that cohort time [26, 27].

Furthermore, our study shows that, over clinical cancer features, also physical factors including BMI and ABO-bgs predicted the risk of PSM in multivariate models, as well. This is an important issue when counseling PCa patients for RARP because the detection of PSM represents an unfavorable pathological outcome that adversely impacts the natural history of the disease for biochemical recurrence, metastatic progression, and disease-specific mortality [20]. Additionally, it is known that obese patients represent a special category that associates with a more challenging surgery with the increased risk of less accurate oncological surgery and increased risk of cancer recurrence and progression after open and or robotic procedures [20, 28–32].

The results of our study might be explained by considering biology of the ABO-bg system that has antigens expressed not only on erythrocytes, but also on epithelial and endothelial cells, as well [3–12]. Such association has been explained by several mechanisms including intercellular adhesion, membrane signaling, angiogenesis, inflammation, and immune surveillance of malignant transformed cells; furthermore, the system may also be related to tumor progression [3–12]. As a theory, the expression of genotype O on PCa cells may influence intercellular adhesion and membrane signaling; furthermore, stimulation of angiogenesis as decreased immunosurveillance may promote tumor growth and extension beyond the prostate capsule thus increasing the risk of PSM.

This study has limitations for being retrospective and for not adjusting for nerve-sparing surgery because such data were not available for all patients. Additionally, data on tumor location were not available. However, it has also strengths for data being collected prospectively and for the population being large and homogenous; additionally, blood groups were all determined at the Department of Transfusion Medicine of our hospital. Furthermore, we have already shown that the risk of biochemical recurrence associated with focal PSM, which closely related to the high-volume surgeon, while nerve-sparing surgery did not have any significant impact [26, 27, 33–35]. As such, our study has also clinical implications for ABO-bgs being an independent predictor of PSM risk.

## Conclusions

Along the ABO-bgs, the risk of PSM was increased by group O independently by other standard preoperative factors. The ABO-bgs may represent a further physical factor for clinical assessment of PCa patients, but confirmatory studies are required.
